# Cooked pork-derived exosome nanovesicles mediate metabolic disorder—microRNA could be the culprit

**DOI:** 10.1186/s12951-023-01837-y

**Published:** 2023-03-09

**Authors:** Linyuan Shen, Jianfeng Ma, Yiting Yang, Tianci Liao, Jinyong Wang, Lei Chen, Shunhua Zhang, Ye Zhao, Lili Niu, Xiaoxia Hao, Anan Jiang, Xuewei Li, Mailin Gan, Li Zhu

**Affiliations:** 1 Farm Animal Genetic Resource Exploration and Innovation Key Laboratory of Sichuan Province, Sichuan Agricultural University, Chengdu, 611130 China; 2 Key Laboratory of Livestock and Poultry Multi-Omics, Ministry of Agriculture and Rural Affairs, College of Animal and Technology, Sichuan Agricultural University, Chengdu, 611130 China; 3 Chongqing Academy of Animal Science, Chongqing, 402460 China

**Keywords:** Meat, Pork, Exosome, microRNA, Transcriptome

## Abstract

In this study, exosomes from cooked meat were extracted by ultra-high-speed centrifugation. Approximately 80% of exosome vesicles were within 20–200 nm. In addition, the surface biomarkers of isolated exosomes were evaluated using flow cytometry. Further studies showed the exosomal microRNA profiles were different among cooked porcine muscle, fat and liver. Cooked pork-derived exosomes were chronically administered to ICR mice by drinking for 80 days. The mice plasma levels of miR-1, miR-133a-3p, miR-206 and miR-99a were increased to varying degrees after drinking exosome enriched water. Furthermore, GTT and ITT results confirmed an abnormal glucose metabolism and insulin resistance in mice. Moreover, the lipid droplets were significantly increased in the mice liver. A transcriptome analysis performed with mice liver samples identified 446 differentially expressed genes (DEGs). Functional enrichment analysis found that DEGs were enriched in metabolic pathways. Overall, the results suggest that microRNAs derived form cooked pork may function as a critical regulator of metabolic disorder in mice.

## Introduction

Extracellular vesicles (EVs) are lipid bilayer membrane vesicles produced by all types of cells in the organism [1]. Despite the continuous development of the field, EVs are currently divided into two categories: ectosomes and exosomes [2]. Exosomes are composed of 40–160 nm-sized vesicles (~ 100 nm on average) [3]. This class is crucial for intercellular communication allowing the substances exchange between cells [4]. Moreover, exosome contents have been considered s potential biomarkers in several diseases, including cancers [5], Parkinson's disease [6], and Alzheimer's disease [7].

It has been described that exosomes are mainly composed of lipids, proteins and nucleic acids (predominantly microRNA (miRNA)) [8]. Interestingly, exosomal miRNA has been widely studied. MicroRNA is a non-coding small RNA presenting a highly conserved mechanism of action. Recently, several studies have suggested that exosomal miRNAs in food can be absorbed through the intestine, playing an essential function in the organism. Xiao et al. A previous report evealed the presence of exosomes in 11 edible fruits and vegetables and successfully identified miRNA profiles in exosomes [9]. Furthermore, exosomal miRNA could be a key factor in functional food. For example, one study demonstrated that ginger-derived exosomes could promote mucosal tissue healing in mice with colitis [10]. In addition, some reports showed that exosome vesicles containing miRNA could be found in milk. Gu et al. reported that the exosomes in pig colostrum contain rich immune related miRNAs [11].

Over the years, pork has been an essential human food source being the most consumed meat worldwide. Usually, the population consumes more cooked meat than raw meat.. Moreover, a previous study established that exosomes and microRNAs are stable at high temperatures [12]. Therefore, this study aimed to identify exosomes in the cooked porcine muscle, fat and liver samples and investigate the microRNA profiles in cooked meat. Furthermore, this study also aimed to determine the effect of feeding exosomes obtained from cooked pork on the growth of mice.

## Materials and methods

### Exosomes isolation

In this study, exosomes were isolated from cooked porcine muscle, fat and liver samples using a previous methodology [13] with some modifications. Briefly, approximately 250 g of meat was minced in a meat grinder, 250 g of water was added, and boiled for 1 h until the meat was fully cooked. Then, the broth was filtered through filter paper and cooled to room temperature. The filtrate was centrifuged at 2,000×*g* for 30 min in an ultracentrifuge (Optima Max-XP, Beckmann Coulter, USA). Then the supernatant was collected, centrifuged for 45 min at 12,000×*g* and filtered through a 0.22 μm filter (Millipore Millex-GP, SLGPR33RB). Next, the filtrate was centrifuged at 160,000×*g* for 2 h, the precipitate was collected and washed with phosphate-buffered saline (PBS), and centrifuged at 160,000×*g* for 1 h. Finally, the precipitate was re-suspended in PBS to obtain exosome suspensions.

### Characterization of cooked meat-derived exosomes

Exosomal morphology was studied using a transmission electron microscope (TEM, HT7800, Hitachi, Japan). Samples were prepared as described previously [14]. Additionally, atomic force microscopy image capture was performed according to a previous methodology [9]. The particle size of exosomes was determined by dynamic light scattering analysis (DLS) using Zetasizer Nano ZS (Malvern Instruments, Worchestershire, UK). The exosome characteristic protein markers CD63 (#557288, BD Pharmingen™, USA) and CD81 (#551108, BD Pharmingen™, USA) were detected using a flow cytometer (BD Accuri C6 Flow Cytomenter). For this assay, exosomes were preincubated with Fc-block (BD Biosciences, CA, USA) before staining to block nonspecific Fc-receptor–mediated binding.

### Mice and feeding

All mice and diets were purchased from Dashuo Company (Chengdu, China). The ICR female mice (∼14 g, 4 weeks old) were randomized into two groups. One group was supplemented with porcine muscle-derived exosomes dissolved in PBS for 80 days (EXD, n = 6). Mice was fed daily with a quantity of exosomes equivalent to the quantity contained in 5 g of pork. In addition, the second group received PBS and was used as a control (CON, n = 6). In this study, the normal drinking water of mice was replaced with PBS or PBS containing exosomes between 9 and 10 a.m. every day.

### Tissue section staining, glucose tolerance test (GTT), and insulin tolerance test (ITT) determination

The hematoxylin and eosin (HE) and Oil Red O stainings were performed according to ourpreviously published methods [15]. Furthermore, GTT and ITT also were performed according to previous methodology [15].

### RNA extraction, library construction, sequencing and RT-qPCR

RNA was isolated using Trizol LS (Ambion, Carlsbad, CA, USA) according to the manufacturer’s instructions. The library construction and sequencing of exosomal miRNA and liver mRNA was performed by Lianchuan BioTech Co., Ltd (Lianchuan Bio, Hangzhou, China). A previous methodology was adopted to perform the RNA-seq analyses [16, 17]. Differentially expressed genes (DEGs) were identified using the edgeR software (|logFC|> 1, *p* value < 0.05).

Reverse transcription-quantitative PCR (RT-qPCR) was performed using Mir-X™ miRNA First Strand synthesis kit (Takara, Dalian, China) and TB Green Premix Ex Taq II kit (Takara, Dalian, China) on a CFX96 system (Bio-Rad, CA, USA). After PCR, absolute quantification was performed by a standard curve method.

### Everted intestinal sac assay

The everted intestinal sac as an in vitro model was prepared following Liu et al. [18]. Female SD rats were fasted overnight and anesthetized to excise jejunum. Each intestinal segment was cut to 4 cm long segments and rinsed with cold saline and immediately placed in 37 °C Tyrode's buffer oxygenated with 95% O_2_ and 5% CO_2_. The intestinal sacs were ligated at one end and gently everted with a glass rod. Exosomes extracted from 250 g meat were added to 20 mL Tyrode's buffer. The control group was conducted without exosomes. The intestinal sac model was incubated in a thermostatic shaker at 37 °C with 50 rpm for 2 h.

### miRNA target gene prediction and functional enrichment analysis

The target gene of exosomal miRNAs were analyzed and predicted by online platform OmicStudio (https://www.omicstudio.cn/analysis) by using *Sus scrofa* and *Mus musculus* as species background, respectively. Gene Ontology (GO) and Kyoto Gene and Encyclopedia of Genomes (KEGG) enrichment analysis were performed using DAVID knowledgebase (v2022q3, https://david.ncifcrf.gov). Protein–protein interaction (PPI) networks were constructed using the STRING database for Cytoscape software 3.9.1.

### Statistical analysis

Data were visualized using GraphPad Prism 8.0 software. The results were represented as mean ± SEM. Comparisons between two groups were performed using a two-tailed Student's *t*-test. *p* < 0.05 was considered statistical significance (*p < 0.05, **p < 0.01).

## Results

### Identification of exosomes isolated from cooked meat

**Fig. 1:**
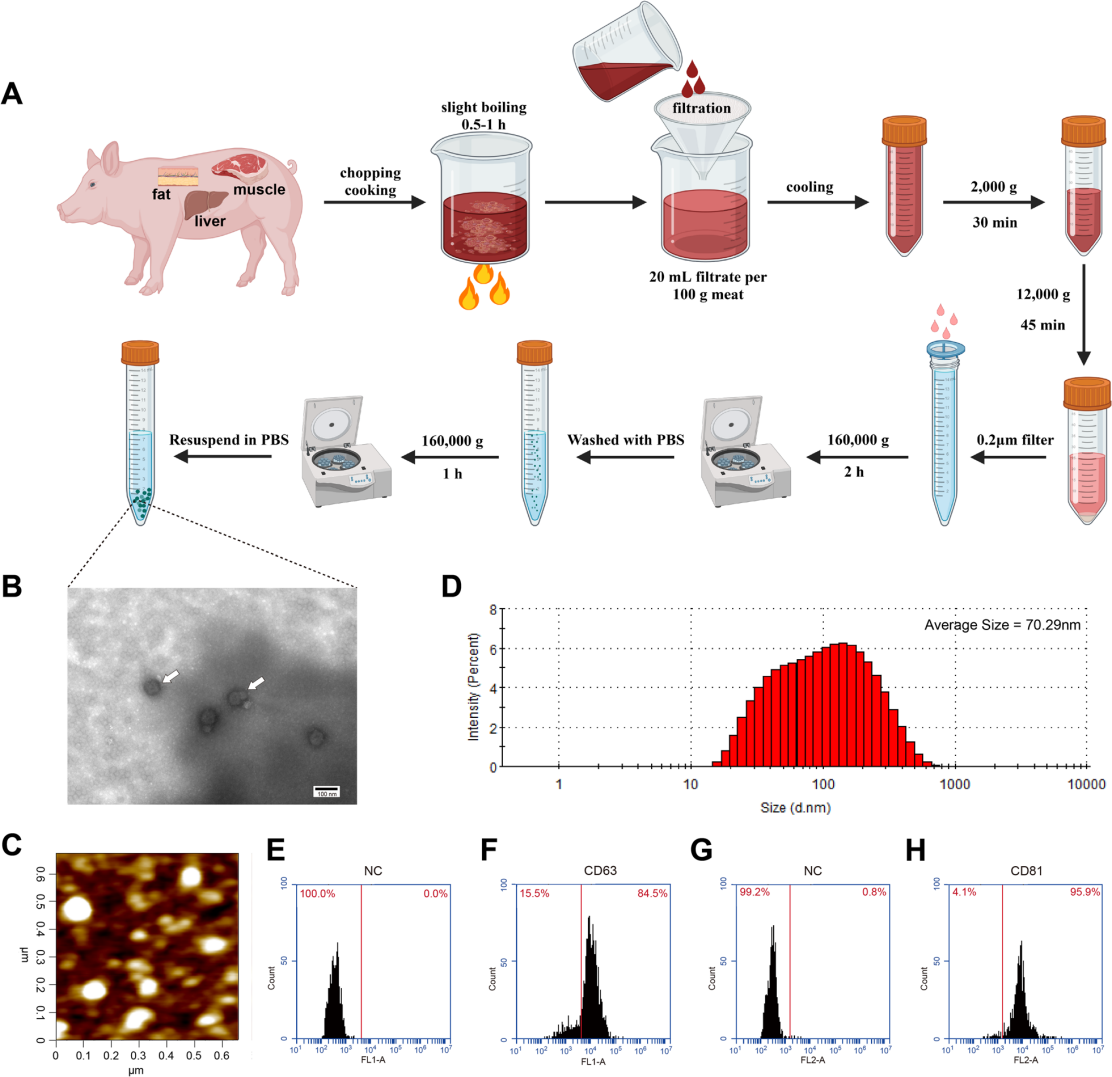
Identification of exosomes isolated from cooked meat. **a** Flow chart of exosomes isolation from cooked porcine muscle, fat and liver, respectively. **b** Representative electron microscope image of CM-Exo. **c** Representative atomic force microscope image of CM-Exo. **d** The size distribution of isolated exosomes by Zetasizer Nano ZS. **e–h** Flow-cytometric analysis of exosome markers CD63 and CD81. Negative controls (NC) were exosome samples to which no antibody was added

**Fig. 2:**
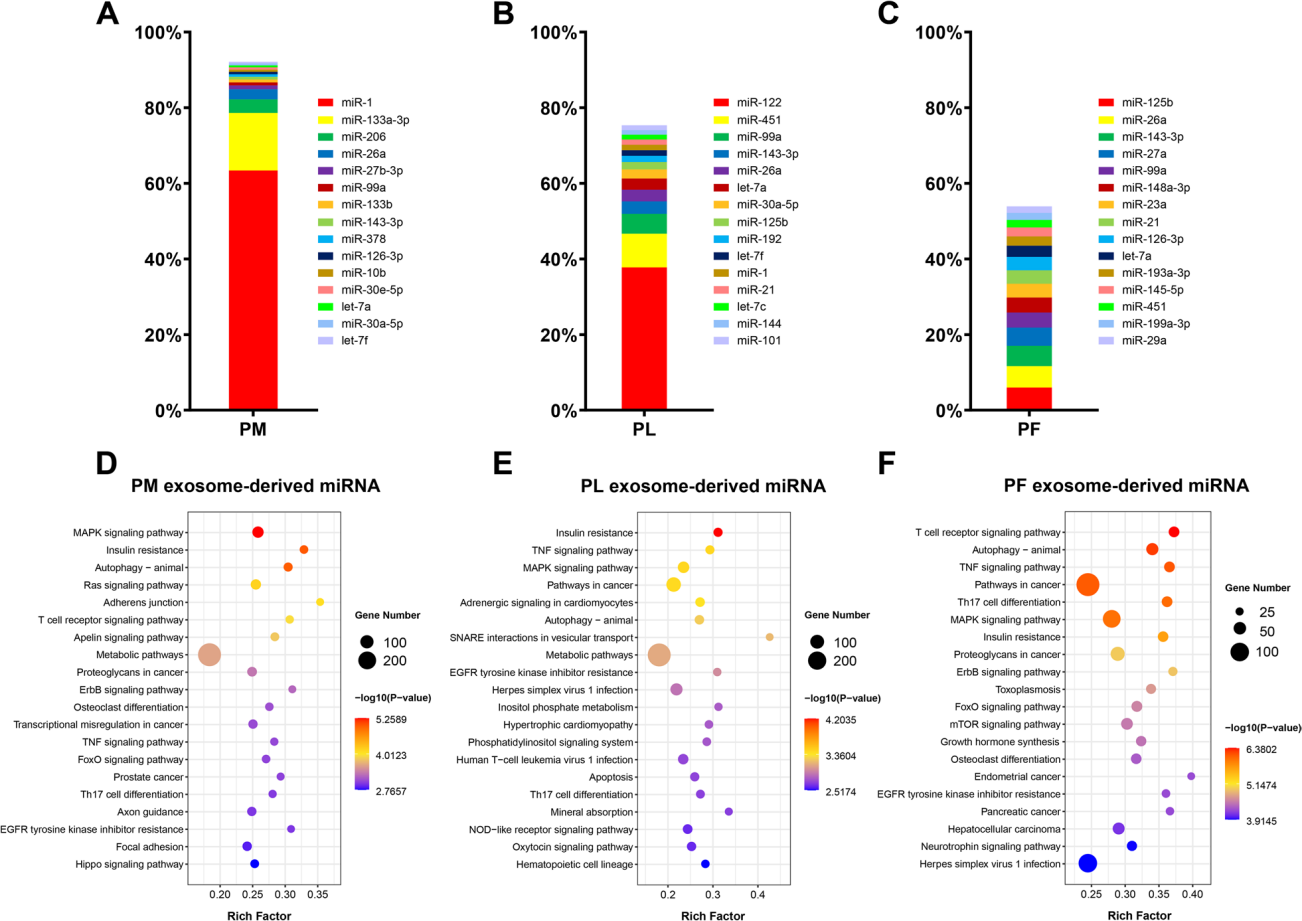
Percentage of the miRNAs in exosomes isolated from PE (**a**), PL (**b**) and PF (**c**). KEGG pathway enrichment analysis of the potential target genes of top ten miRNAs in PME (**d**), PLE (**e**) and PFE (**f**), respectively

In this study, exosomes were isolated from cooked meat (CM-Exo), including porcine muscle (PME), fat (PFE) and liver (PLE) (Fig. 1a). The purified samples were further characterized by TEM and atomic force microscopy (AFM). The vesicles with exosome-like features were confirmed in cooked meat (Fig. 2b, c). Moreover, the average particles size of those exosome-like vesicles showed 70.29 nm, and approximately 80% were within 20–200 nm (Fig. 2d). Furthermore, flow cytometry results demonstrated that the positive rates of exosome-specific markers CD63 and CD81 were 84.5% (Fig. 1e, f) and 95.9% (Fig. 1g, h), respectively.

### microRNA profiles of CM-Exo

The miRNA expression profile in PME, PLE and PFE samples was investigated by high throughput sequencing. The results revealed that miR-1, miR-133a-3p and miR-206 contribute to more than 80% of the total miRNA in the PME sample (Fig. 2a). The relative abundance of miR-122 (37.8%) was highest in the PLE sample, followed by miR-451 (8.9%), miR-99a (5.3%) and others miRNAs (Fig. 2b). On the other hand, in the PFE sample, miR-125b, miR-26a, miR-143-3p, miR-27a and miR-99a were the most abundant (Fig. 2c). Furthermore, a KEGG pathway enrichment analysis revealed that miRNAs obtained from the PME sample were mainly involved in the MAPK signaling pathway, insulin resistance and autophagy (Fig. 2d). Additionally, the top three significantly enriched pathways for the miRNAs derived from PLEwere insulin resistance, TNF and MAPK signaling pathway (Fig. 2e). Moreover, the T cell receptor signaling pathway, autophagy and TNF signaling pathway were the most significantly represented factors in miRNAs obtained from PFE (Fig. 2f).

### Effect of supplementary feeding cooked pork-derived exosomes on growth of mice

**Fig. 3:**
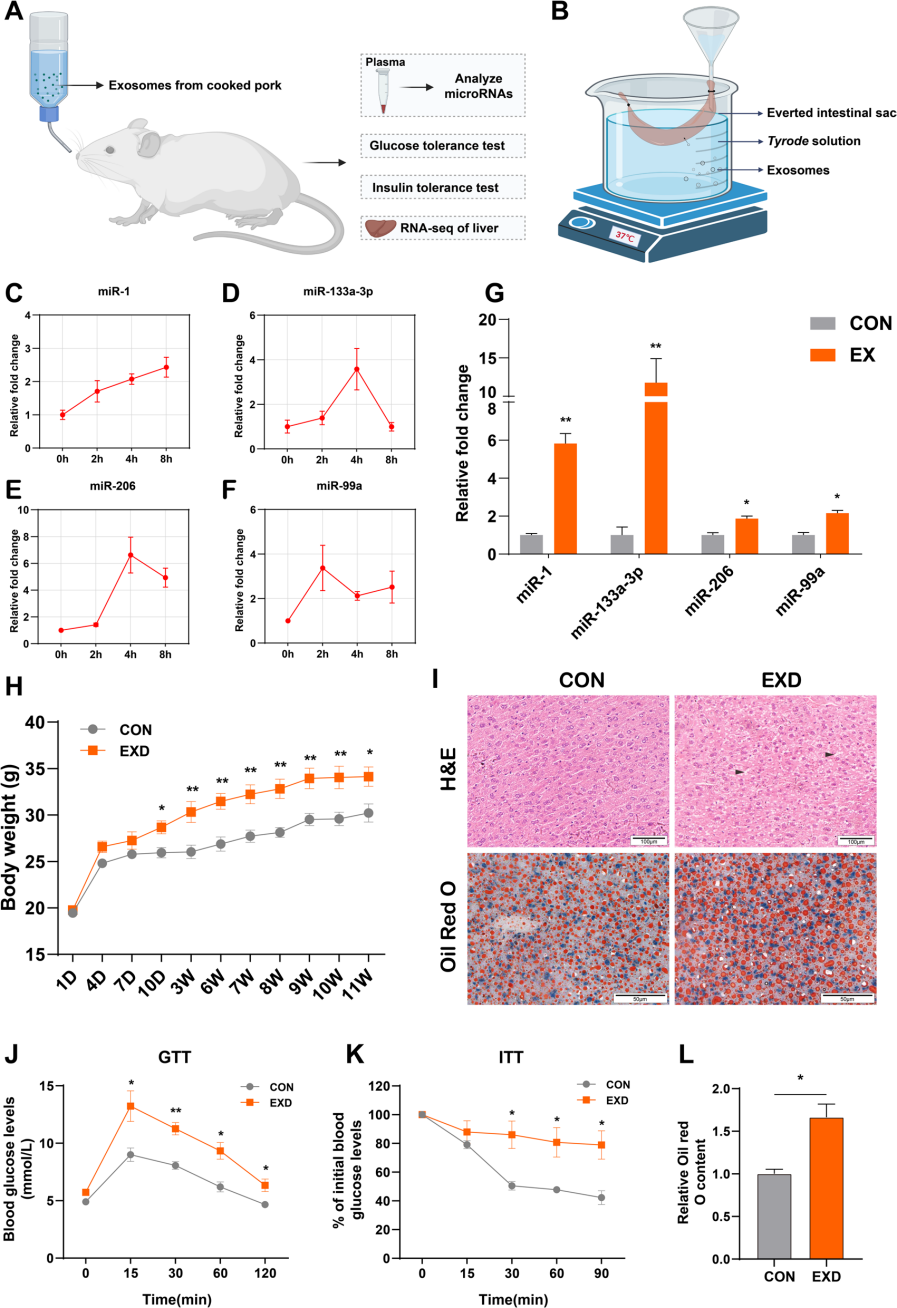
Mice were given drinking water supplemented with PME. **a** A schematic showing the experimental procedure. **b** Everted intestinal sac assay schematic. **c–f** Fold change in plasma miRNA relative to initial levels. **g** Fold change of miRNA in everted intestinal sac (EX) relative to control group (CON). **h** Body weights of mice (n = 6). D: day. W: week. **i** HE and Oil Red O staining of the liver. **j** Glucose tolerance test (GTT). **k** Insulin tolerance test (ITT). **l** Quantification of the Oil Red O staining images. EXD: mice supplemented exosomes in drinking water. CON: mice supplemented PBS in drinking water. Results are presented as mean ± SE (n = 3 except as indicated otherwise, *p < 0.05, **p < 0.01)

To evaluate the effects of cooked pork-derived exomes on the growth of mice, exosomes (equivalent to the quantity contained in 5 g of pork) were added to the drinking water daily for up to 80 days (Fig. 3a). After the exosomes intake, we assessed several miRNAs levels in mice plasma with a higher relative abundance in exosomes (Fig. 3c–f). The results showed that the miR-1, miR-133a-3p, miR-206 and miR-99a levels increased within 0–8 h in mice after exosome ingestion. In addition, the everted intestinal sac assay was performed (Fig. 3b). A significant increase in the four miRNAs level in everted intestinal sac was observed after adding exosomes to the buffer (Fig. 3g).

This study also evaluated the body weight of mice after ingestion of exosomes. The results demonstrated a significant increase in the exosome-supplemented mice group compared to the CON group after 10 days of ingestion (Fig. 3h). Following intraperitoneal glucose injection, the EXD group exhibited significantly higher blood glucose levels at 15–120 min compared to the CON group (Fig. 3j). Additionally, the ITT results showed impaired blood glucose consumption and reduced insulin sensitivity in the EXD group (Fig. 3k). In addition, the histomorphological analysis performed in the liver showed small vacuoles in the EXD group (Fig. 3i). On the other hand, the liver of the EXD group contained more abundant lipid droplets compared to the CON group (Fig. 3l).

### Cooked pork-derived exosome cause transcriptome changes in mice liver

**Fig. 4:**
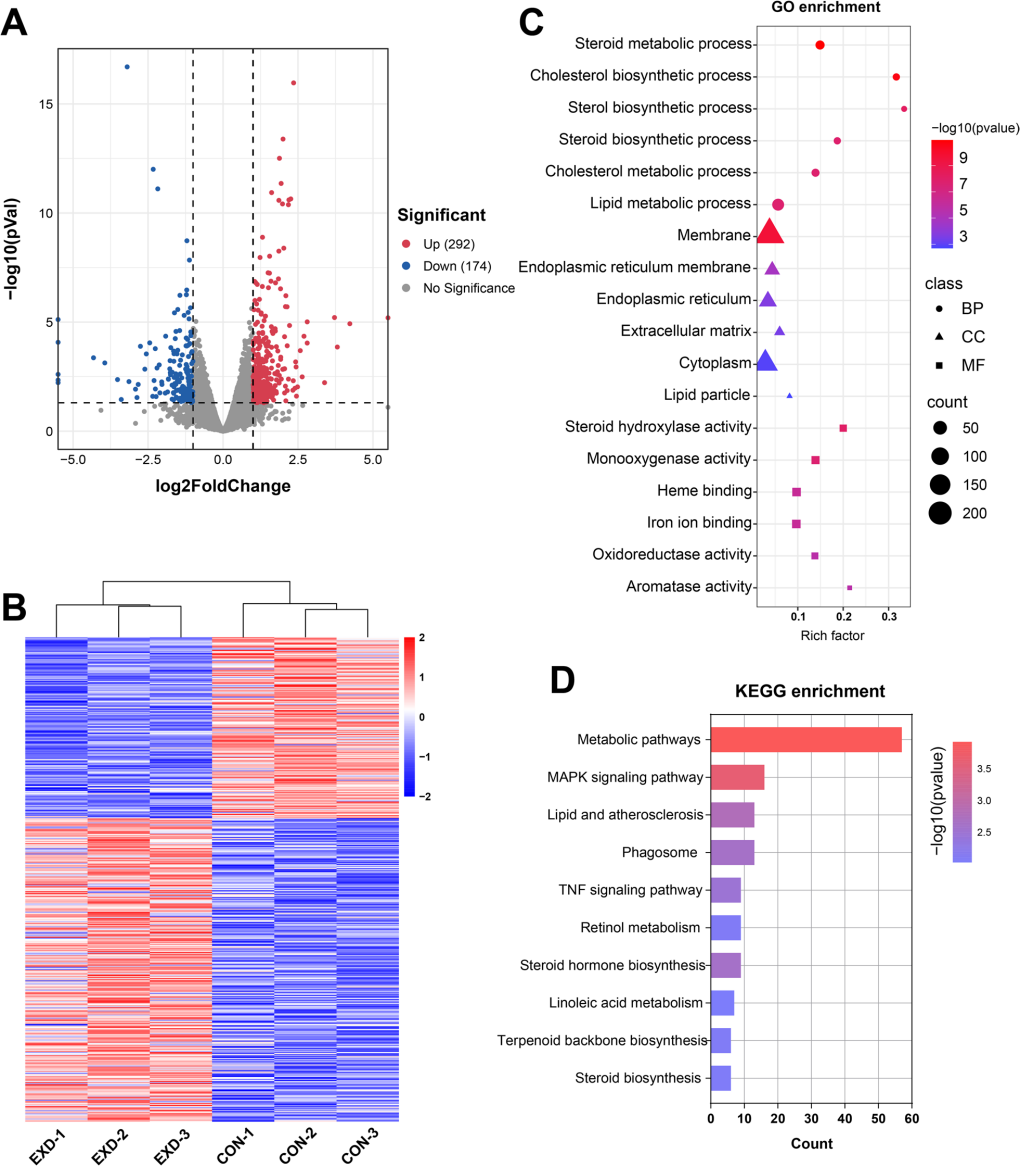
The overview of the transcriptomic changes of mouse liver. **a** Volcano plot of differential gene expression. **b** EXD group and CON group difference gene clustering analysis heatmap. **c** GO enrichment analysis of DEGs. **d** KEGG enrichment analysis of DEGs

Furthermore, the liver transcriptomic changes were evaluated after the ingestion of exosomes. Overall, 466 DEGs with 292 genes upregulated and 174 genes downregulated were identified in the EXD group (Fig. 4a). Heatmap presenting DGEs in EXD and CON groups are shown in Fig. 4b. The GO enrichment analysis revealed that DEGs were mainly enriched in the steroid metabolic process, sterol and steroid biosynthetic process, cholesterol and lipid metabolic process (Fig. 4c). Furthermore, the KEGG enrichment analysis showed the DEGs were mainly enriched in metabolism pathways, MAPK signaling pathway, lipid and atherosclerosis (Fig. 4d).

### Potential regulatory networks involved in lipid metabolic process

**Fig. 5:**
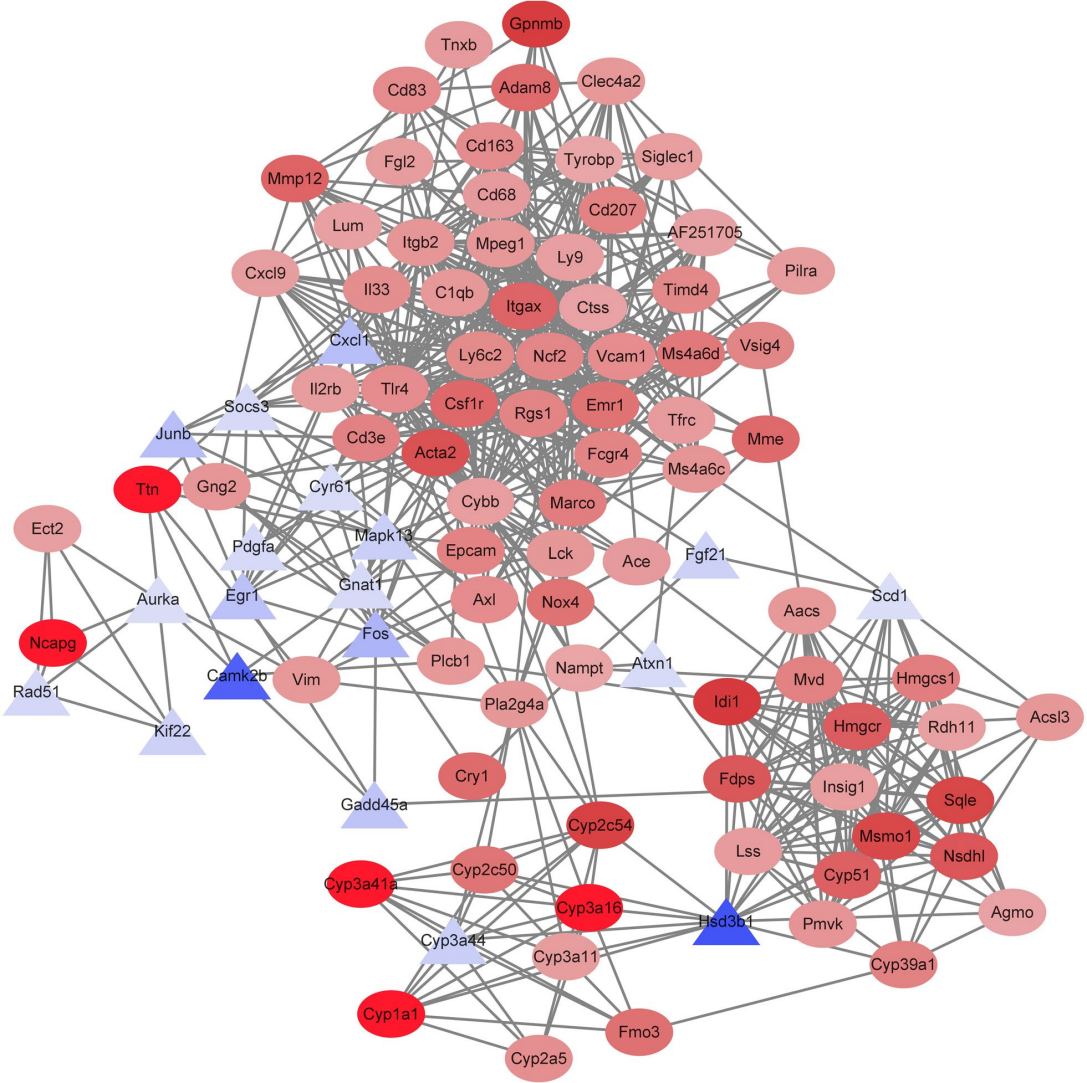
Top 100 hub genes identified from the PPI network. Each node represents a hub gene. Red ovals represent upregulated genes, blue triangle represented downregulated genes. The color depth represents the fold-change of hub genes

**Fig. 6:**
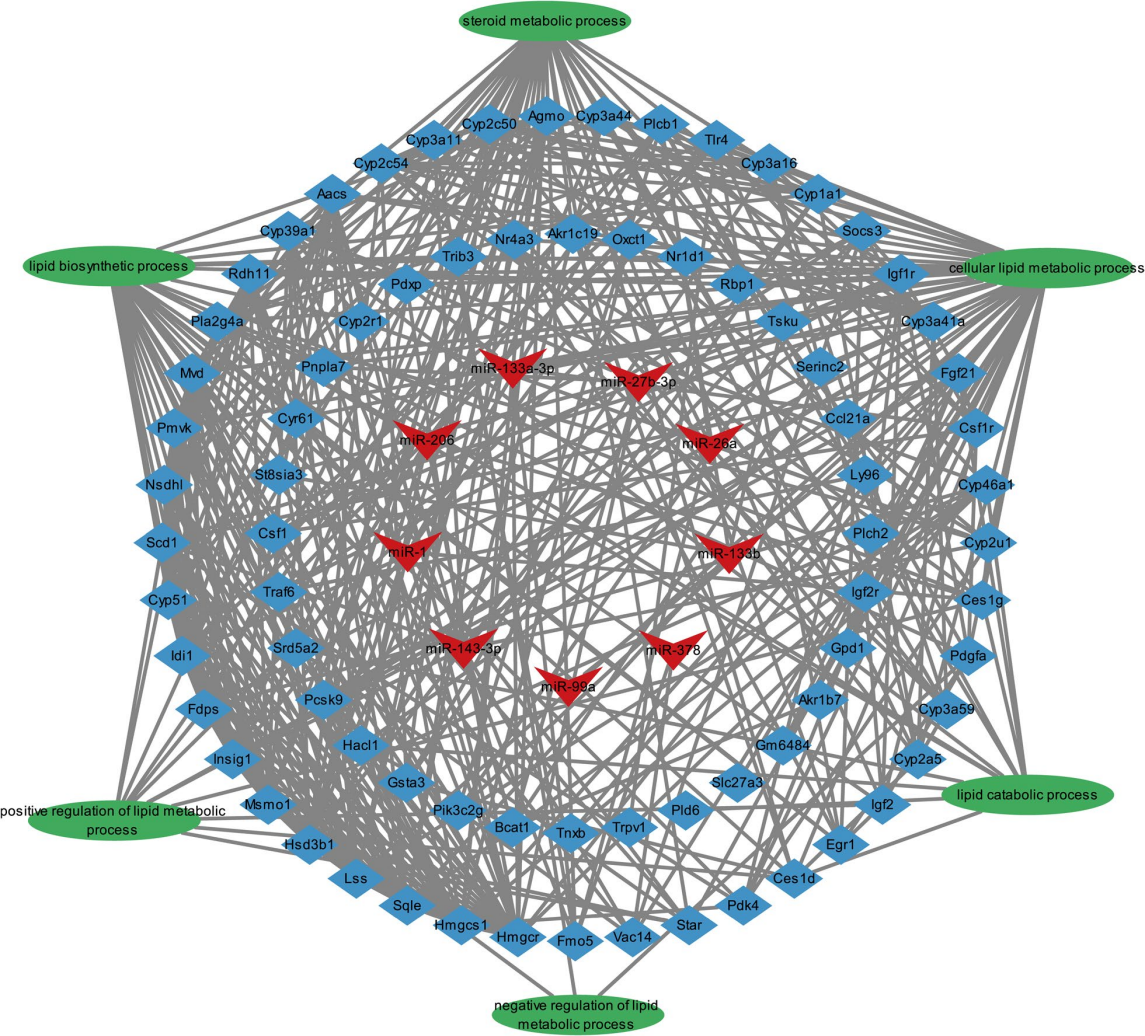
miRNA–mRNA–lipid metabolism pathways regulatroy network. The red node represents miRNA. Blue nodes represent target genes. Green node represents lipid metabolism pathway

In this study, a PPI network including the top 100 hub genes was constructed (Fig. 5), and the results showed that *Itgax*, *Tlr4*, *Itgb2*, *Csf1r* and *Tyrobp* were the top hub genes. Taking into consideration the altered lipid metabolism (Fig. 3), a potential miRNA–mRNA–lipid metabolism pathways regulatory network was constructed (Fig. 6), and a significant interconnection between exosomal miRNA and liver lipid metabolism pathways was detected.

## Discussion

Current studies have shown that exosomes are widely found in foodstuffs, including vegetables, fruits [9], and milk [19]. However, the presence and function of exosomes in meat products remain unclear. Several reports revealed that exosomes are present in the muscle and fat of animals [20]. The biogenesis process of exosome involves the plasma membrane invagination and the formation of intracellular multivesicular bodies [3]. Therefore, this indicates that exosome-like vesicles are widespread in cells before the exosomes secretion. In this study, cooked meat-derived exosomes were obtained and identified.. CM-exo was evaluated using TEM and AFM imaging (Fig. 2b, c). The results revealed that the average diameter of the CM-exo was 70.29 nm. Similarly to our results, a previous study also reported that the average size of exosomes extracted from milk was 75.7 nm [21]. However, plant-derived exosome nanoparticles have a diameter of approximately 50–500 nm [22], indicating that exosomes obtained form animals or plants could be distinct. Moreover, relative high positivity rates for CD63 and CD81 were detected. Therefore, these results suggest that exosomes are present and can be isolated from cooked meat.

The exosome mainly contain lipids, proteins and nucleic acids, predominantly microRNA (miRNA) [8]. Recently, some studies indicated that exosomal miRNA in food could be absorbed via the gut having crucial biological functions in the organism. Therefore, we evaluated the miRNA expression profile in CM-exo through miRNA sequencing. We observed great differences in exosomal miRNA cargo in muscle, fat and liver samples. For example, in the PME samples, miR-1 and miR-133a-3p were predominant, while miR-122 and miR-451 were present in higher abundance for the PLE sample. Subsequently, we perform miRNA functional enrichment analysis for the top 10 miRNAs. Interestingly, PME and PLE miRNAs were found to be significantly enriched with insulin resistance. Meanwhile, metabolic pathways had the highest number of enriched target genes. Previous reports indicated that exosomes modulate several biological processes, including chronic inflammation, insulin resistance and metabolic disorders [23]. Therefore, we further investigated the effect of cooked meat-derived exosomes on metabolism of mice.

Some highly-expressed exosomal miRNAs were altered in plasma after oral administration of drinking water containing exosomes. The levels of miR-1, miR-133a-3p, miR-206 and miR-99a were increased to varying degrees in mice plasma. As observed in the present study, milk-derived exosomal miRNA could be absorbed by the organism [24]. In our study, everted intestinal sac assay also confirmed this. Interestingly, the mice group supplemented with PME exhibited significantly faster growth compared to the control group. Additionally, abnormal glucose metabolism and insulin resistance were detected in the EXD group. Moreover, the liver samples analysis revealed that lipid droplets were significantly increased in the ECD group. The liver is a major metabolic organ crucial during lipids and glucose metabolism [25]. The transcriptomic results obtained in this study can comfirm this information. DEGs in the liver were predominantly enriched in metabolic pathways. Furthermore, the PPI network analysis suggested that DEGs could be divided into three clusters. We observed numerous genes linked to lipid metabolism were centrally located, such as *Insig1* [26], *Tlr4* [27], *Scd1* [28]. The relative abundance of miR-1 in PME was the highest. It has been described that miR-1 can induce lipogenesis in hepatocytes [29]. On the other hand, miR-143-3p exhibited the maximum number of target genes in network (Fig. 6). Previous studies showed that miR-143-3p could be an essential regulator of insulin resistance and lipid deposition in mice liver metabolic syndrome [30]. Therefore, the miRNA–mRNA–pathway regulatory network indicated that the PME miRNA could be a critical regulator involved in the metabolic disorder of mice.

## Conclusion

Overall, this study demonstrated that exosomes could be detected in cooked meat products, including porcine muscle, fat and liver, and differences in exosomal miRNA were detected between these tissues. Moreover, the mice supplemented with long-term cooked-pork-derived exosomes exhibited insulin resistance and lipid metabolic disorder in the liver. Additional research is needed, and ongoing, to determine which active ingredients of exsomes play a role in dysmetabolism. This work suggested that exosomes derived from meat products could not be ignored, where miRNA may not be beneficial for our health.

## Data Availability

The datasets used and analysed during the current study are available from the corresponding author on reasonable request.

## References

[1] Raposo G, Stoorvogel W (2013). Extracellular vesicles: exosomes, microvesicles, and friends. J Cell Biol.

[2] Meldolesi J (2018). Exosomes and ectosomes in intercellular communication. Curr Biol.

[3] Kalluri R, LeBleu VS. The biology, function, and biomedical applications of exosomes. Science. 2020;367:eaau6977. 10.1126/science.aau6977 PMC7717626 32029601

[4] Dolcetti E, Bruno A, Guadalupi L, Rizzo FR, Musella A, Gentile A (2020). Emerging role of extracellular vesicles in the pathophysiology of multiple sclerosis. Int J Mol Sci.

[5] Ebrahimi N, Faghihkhorasani F, Fakhr SS, Moghaddam PR, Yazdani E, Kheradmand Z (2022). Tumor-derived exosomal non-coding RNAs as diagnostic biomarkers in cancer. Cell Mol Life Sci.

[6] Nila IS, Sumsuzzman DM, Khan ZA, Jung JH, Kazema AS, Kim SJ (2022). Identification of exosomal biomarkers and its optimal isolation and detection method for the diagnosis of Parkinson’s disease: a systematic review and meta-analysis. Ageing Res Rev.

[7] Abner EL, Elahi FM, Jicha GA, Mustapic M, Al-Janabi O, Kramer JH (2020). Endothelial-derived plasma exosome proteins in Alzheimer’s disease angiopathy. FASEB J.

[8] Li D, Liu J, Guo B, Liang C, Dang L, Lu C (2016). Osteoclast-derived exosomal miR-214-3p inhibits osteoblastic bone formation. Nat Commun.

[9] Xiao J, Feng S, Wang X, Long K, Luo Y, Wang Y (2018). Identification of exosome-like nanoparticle-derived microRNAs from 11 edible fruits and vegetables. PeerJ.

[10] Zhang M, Viennois E, Prasad M, Zhang Y, Wang L, Zhang Z (2016). Edible ginger-derived nanoparticles: a novel therapeutic approach for the prevention and treatment of inflammatory bowel disease and colitis-associated cancer. Biomaterials.

[11] Gu Y, Li M, Wang T, Liang Y, Zhong Z, Wang X (2012). Lactation-related microRNA expression profiles of porcine breast milk exosomes. PLoS ONE.

[12] Pieters BCH, Arntz OJ, Bennink MB, Broeren MGA, van Caam APM, Koenders MI (2015). Commercial cow milk contains physically stable extracellular vesicles expressing immunoregulatory TGF-β. PLoS ONE.

[13] Palanisamy V, Sharma S, Deshpande A, Zhou H, Gimzewski J, Wong DT (2010). Nanostructural and transcriptomic analyses of human saliva derived exosomes. PLoS ONE.

[14] Wei Y, Wang D, Jin F, Bian Z, Li L, Liang H (2017). Pyruvate kinase type M2 promotes tumour cell exosome release via phosphorylating synaptosome-associated protein 23. Nat Commun.

[15] Shen L, He J, Zhao Y, Niu L, Chen L, Tang G (2021). MicroRNA-126b-5p exacerbates development of adipose tissue and diet-induced obesity. Int J Mol Sci.

[16] Gan M, Liu L, Zhang S, Guo Z, Tan Y, Luo J (2021). Expression characteristics of microRNA in pig umbilical venous blood and umbilical arterial blood. Animals.

[17] Tan Y, Gan M, Shen L, Li L, Fan Y, Chen Y (2021). Profiling and functional analysis of long noncoding RNAs and mRNAs during porcine skeletal muscle development. Int J Mol Sci.

[18] Liu H, Tu L, Zhou Y, Dang Z, Wang L, Du J, et al. Improved bioavailability and antitumor effect of docetaxel by TPGS modified proniosomes: in vitro and in vivo evaluations. Sci Rep. 2017;7:43372. 10.1038/srep43372 PMC5339906 28266539

[19] Li D, Yao X, Yue J, Fang Y, Cao G, Midgley AC (2022). Advances in bioactivity of microRNAs of plant-derived exosome-like nanoparticles and milk-derived extracellular vesicles. J Agric Food Chem.

[20] Rome S (2022). Muscle and adipose tissue communicate with extracellular vesicles. Int J Mol Sci.

[21] Yun B, Maburutse BE, Kang M, Park MR, Park DJ, Kim Y (2020). Short communication: dietary bovine milk-derived exosomes improve bone health in an osteoporosis-induced mouse model. J Dairy Sci.

[22] Kim J, Li S, Zhang S, Wang J (2022). Plant-derived exosome-like nanoparticles and their therapeutic activities. Asian J Pharm Sci.

[23] Yao Z-Y, Chen W-B, Shao S-S, Ma S-Z, Yang C-B, Li M-Z (2018). Role of exosome-associated microRNA in diagnostic and therapeutic applications to metabolic disorders. J Zhejiang Univ Sci B.

[24] Wang L, Sadri M, Giraud D, Zempleni J (2018). RNase H2-dependent polymerase chain reaction and elimination of confounders in sample collection, storage, and analysis strengthen evidence that microRNAs in bovine milk are bioavailable in humans. J Nutr.

[25] Yoshida H, Tsuhako R, Atsumi T, Narumi K, Watanabe W, Sugita C (2017). Naringenin interferes with the anti-diabetic actions of pioglitazone via pharmacodynamic interactions. J Nat Med.

[26] Xu Y, Tao J, Yu X, Wu Y, Chen Y, You K (2021). Hypomorphic ASGR1 modulates lipid homeostasis via INSIG1-mediated SREBP signaling suppression. JCI Insight.

[27] Pang S, Tang H, Zhuo S, Zang YQ, Le Y (2010). Regulation of fasting fuel metabolism by toll-like receptor 4. Diabetes.

[28] Olichwier A, Balatskyi VV, Wolosiewicz M, Ntambi JM, Dobrzyn P (2020). Interplay between thyroid hormones and stearoyl-CoA desaturase 1 in the regulation of lipid metabolism in the heart. Int J Mol Sci.

[29] Zhong D, Huang G, Zhang Y, Zeng Y, Xu Z, Zhao Y (2013). MicroRNA-1 and microRNA-206 suppress LXRα-induced lipogenesis in hepatocytes. Cell Signal.

[30] Lin X, Du Y, Lu W, Gui W, Sun S, Zhu Y (2021). CircRNF111 protects against insulin resistance and lipid deposition via regulating miR-143-3p/IGF2R axis in metabolic syndrome. Front Cell Dev Biol.

